# Internal state effects on behavioral shifts in freely behaving praying mantises (*Tenodera sinensis*)

**DOI:** 10.1371/journal.pcbi.1009618

**Published:** 2021-12-20

**Authors:** Shanel C. Pickard, David J. Bertsch, Zoe Le Garrec, Roy E. Ritzmann, Roger D. Quinn, Nicholas S. Szczecinski

**Affiliations:** 1 Department of Mechanical and Aerospace Engineering, Case Western Reserve University, Cleveland, Ohio, United States of America; 2 Department of Biology, Case Western Reserve University, Cleveland, Ohio, United States of America; 3 Department of Mechanical and Aerospace Engineering, West Virginia University, Morgantown, West Virginia, United States of America; University of California Santa Barbara, UNITED STATES

## Abstract

How we interact with our environment largely depends on both the external cues presented by our surroundings and the internal state from within. Internal states are the ever-changing physiological conditions that communicate the immediate survival needs and motivate the animal to behaviorally fulfill them. Satiety level constitutes such a state, and therefore has a dynamic influence on the output behaviors of an animal. In predatory insects like the praying mantis, hunting tactics, grooming, and mating have been shown to change hierarchical organization of behaviors depending on satiety. Here, we analyze behavior sequences of freely hunting praying mantises (*Tenodera sinensis*) to explore potential differences in sequential patterning of behavior as a correlate of satiety. First, our data supports previous work that showed starved praying mantises were not just more often attentive to prey, but also more often attentive to *further* prey. This was indicated by the increased time fraction spent in attentive bouts such as prey monitoring, head turns (to track prey), translations (closing the distance to the prey), and more strike attempts. With increasing satiety, praying mantises showed reduced time in these behaviors and exhibited them primarily towards close-proximity prey. Furthermore, our data demonstrates that during states of starvation, the praying mantis exhibits a stereotyped pattern of behavior that is highly motivated by prey capture. As satiety increased, the sequenced behaviors became more variable, indicating a shift away from the necessity of prey capture to more fluid presentations of behavior assembly.

## Author summary

Animals have the incredible ability to adapt behavior to the task at hand. Such an ability is born, in part, from the biochemical modulation of neural networks to contextually integrate sensory information and formulate appropriate behavioral responses. Satiety hormones—and more specifically insulin—have this modulatory effect on various brain regions via strikingly conserved mechanisms across the animal phyla. Like vertebrates, invertebrates have shown impressive abilities to thrive in diverse environments—showing sophisticated adaptive abilities that make these animals more than the “lowly animals” their reputation would make you believe. In this work, the effect of satiety on output behavior of freely hunting mantises was assessed. It was found that in food deprived states, mantises exhibited more stereotyped behavior sequences, with the primary motivation of prey capture. Conversely, as satiety increased, behavior sequences became more flexible. This work suggests that behavior assembly takes on a more rigid structure in times of desperation, when survival of the animal is at stake. The increase in satiety correlates to a change in the hierarchical schema of behavior such that when food is secured, behavior takes a more free-form structure.

## 1 Introduction

Environmental inputs provide a multitude of cues that help drive the appropriate output behaviors of an animal. In a simplified process flow, high dimensional external information is brought into the nervous system via sensors (e.g. visual, olfactory, tactile) and conducted to the deeper perception layers of the brain [1–11]. These centralized neural structures integrate sensory cues across modalities, generating feature determinants that drive decision making and behavioral outputs that are 1) appropriately responsive to the environment and 2) relevant to the current task [2, 3, 5, 12–15]. To add to this complexity is the influence of the animal’s dynamic physiological state—known as the internal state—which is responsible for giving definition to the current motivation [16]. In conjunction with the integrated sensory cues from the environment, motivation influences moment-to-moment, goal-orientated behavior [11, 17, 18]. *How* internal states influence the neural processes that direct behavior is unknown, but regardless of mechanism, they have been identified as a major contributor in instigating behavioral shifts [3, 9, 10, 19, 20].

### 1.1 Satiety effects in vertebrates and invertebrates

Satiety signalling is largely an evolutionarily conserved process with a myriad of participating hormones responsible for upregulating or suppressing food seeking behavior. The complex interplay of these hormones act both locally in gut circuits and in the brain, where it can have far reaching effects via descending commands [20–26]. Of these hormones, a key regulator in satiety-influenced behavioral is insulin (and insulin-like peptide). Its role in metabolic regulation has been exhaustively studied in mammals—including humans—with special attention paid to its role in metabolic disorders like diabetes and obesity [15, 27–29].

In addition to its role in energy regulation, insulin’s neuromodulatory effects are becoming more apparent [9, 15, 20, 30, 31]. In rats, for instance, insulin receptors (IR) have been found diffusely throughout the brain, with particularly high concentrations in the olfactory bulb (OB) and the hypothalamus [32–35]. Long periods of starvation cause downregulation of IR densities on the primary olfactory afferents, without a corresponding decrease of IRs of the hypothalamus [36], suggesting differing roles of insulin within these two substrates; one role being a hormone that communicates metabolic status to the hypothalamus, while the second is serving as a neuromodulator to adjust the gain of olfactory sensory input. Aime *et al*. showed that insulin can act directly on the olfactory bulb to decrease olfactory detection, and in turn, result in decreased sniffing behavior [9]. Additionally, satiety and insulin modulation has been implicated in auditory sensory processing [37], memory formation [38], and anxiety and depression [39, 40].

To date, insulin receptors in arthropods have been found in a multitude of tissues, ranging from the gut to reproductive genitalia—exemplifying the diverse role of insulin in energy management, reproductive proficiency, growth, metabolism, and lifespan [41–44]. As in vertebrates, insulin has far reaching effects within the arthropod brain, modulating olfaction sensation [24, 45], circadian cycle [46], and locomotion [47]. In *Drosophila*, for example, increased satiety was found to reduce olfactory signal transduction directly through insulin modulation. Damped signaling to the deeper integration neuropils, such as the mushroom bodies and the central complex, ultimately led to reduced food seeking behavior [24, 45, 47].

### 1.2 Previous work on satiety in behavior of praying mantises

As in nearly all animals, praying mantises exhibit a large repertoire of behaviors that range in complexity from reflexive responses to organized sequences of behavior [19, 24, 48, 49]. Furthermore, they have the dual roles of being both predator and prey which presents interesting opportunities to study shifts in goal-oriented behavior—especially in the context of behavioral hierarchies [19, 50]. In the role as predator, praying mantises exhibit a diverse approach to hunting that spans from active pursuit to ambush predatory tactics. Previous work has suggested that the internal state (i.e. nutritional status) plays a major role in providing state-dependent pressure to shift between behavioral hunting tactics—with the aggressive pursuit seen in starved animals gradually transitioning into ambush predation as satiety increases [19, 20, 48]. Bertsch et al. showed a correlation between the metabolic state of the animal and the overall area that elicited prey-directed attention. Other behaviors such as head and body orientation, pursuit, and striking at prey significantly decreased with the consumption of food [20]. The authors also showed that injection of insulin was sufficient to change the behavior from active pursuit to “sit-and-wait” ambush, thus implicating this peptide hormone as a metabolic indicator of internal state.

Sated praying mantises are less attentive to distant cockroach prey (⩾15 cm), confining their attention to close opportunistic encounters (≈ 5 cm). Furthermore, sated animals are less likely to initiate locomotor pursuit and had reduced prey capture attempts. However, it has yet to be demonstrated that the suite of predatory behaviors are assembled into different, complex hunting strategies affected by the satiety condition. Here, we quantify the predatory regime seen in the aggressive pursuit strategy and observe how it changes as the satiety condition of the animal is increased over time. We do this through first showing trends of time utilization deployed by praying mantises in each of the five nutritional states (zero to four prey items consumed)—allowing us to define a continuum of behaviors as the nutritional status of the animal changes. Secondly, with the use of sequential analytical approaches, we show that behavior sequence structure is a correlate of internal state. To the best of the authors’ knowledge, no previous study has explored the correlation of satiety to sequential assembly of behavior sequences or the exhibited transition probabilities between hunting and nonhunting regimes of freely behaving mantises.

## 2 Methods

### 2.1 Praying mantis rearing and colony maintenance

Experiments were all conducted exclusively with female *T. sinensis*, 14–18 days post-eclosion. Animals were raised individually in 1.8 liter plastic containers stored at 27°C and exposed to light cycling of 12 hours light:12 hours dark. Animals selected for experiments appeared healthy, with intact limbs and antennae. Chosen animals were permitted water but deprived of food 5-7 days prior to the commencement of experiments. All experimental animals were treated appropriately, and we operated in accordance with ethical animal care guidelines established by our lab.

### 2.2 Arena hunting and video capture

When mantises reached the designated state of starvation that was outlined in section 2.1, hunting experiments were initiated. To begin, a single praying mantis was centrally placed in the experimental arena (37 x 29 cm) followed by four cockroach nymphs *Blaberus discoidalis*); all selected to have a group mean mass of about 190 mg). The arena was underlit to project the silhouettes of both the mantis and nymphs which was filmed overhead with a video camera (Casio Exilim or Point Grey Flea3 camera; video capture done at 29.97 fps). After four cockroach nymphs were placed in the arena, the mantis was permitted to freely hunt for approximately 7 minutes. Within that time, if the praying mantis successfully captured a nymph, the experimenter gently removed the cockroach from the raptorial legs and placed both animals back into the arena. This interruption by the experimenter is what we are designating an “arena reset.” This was done to keep the animal in the same feeding state throughout the experimental run. A cockroach nymph captured towards the end of the allotted 7 minutes was permitted to be consumed by the praying mantis and served as the transition to the new feeding state for the next iteration of the experiment. For example, if the praying mantis was in the 0-fed state and has now consumed 1 cockroach, the mantis is now in the 1-fed state when going into the second trial. After consuming the nymph, the mantis is permitted a 15 minute rest period before being placed back into the arena with four new nymphs, and again, allotted 7 minutes to hunt in the new feeding state. This was repeated with each consecutive experiment having the praying mantis consuming one nymph and increasing in the feeding state—with the last experimental run being a 4-fed state.

All animals (6 mantises) followed the same feed state progression, with the initial state being the starved state (0-fed). Feed status was incrementally increased by the consumption of one nymph at the completion of each experimental trial, with the progression from 0-fed, to 1-fed, and so on through the 4-fed state; all experimental trials for a single animal were completed in the same day to maintain consistent timing between feed states. Because the strict rearing schedule was designed to have the praying mantises in the same maturation stage and feeding state at the commencement of experiments, the order of feeding could not be randomized. The resulting data set was composed of 30 videos (6 mantises, 1 video per feed state, 5 feed states), with each video containing a variable number of subsequences depending on how many arena resets occurred (see Table 1 for summary.)

**Table 1:** Summary of statistics and counts. For each animal, a single video captures behavior while the animal is in a single feed state (5 total videos *per animal* across the 5 feed states). Each video has a variable number of subsequences depending on the number of arena resets that occurred within that video. (A) Summary of the number of subsequences seen for each animal in each feed state. (B) Summary of behavior probabilities presented in section 3.2.1. (C) Summary of bout times for each behavior presented in 3.2.2. (D) Summary of transition count into each behavior by feed state. (E) Summary of miscellaneous metrics that include the average length of subsequences for each feed states, miss strike count, successful strike rate (number of successful prey captures divided by total number of prey capture attempts), number of sequences that end with prey capture, and number of escape attempts by feed state. C and E are reported as *μ*(STD).

**A**	**Number of Subsequences**							
**State**	**Animal 1**	**Animal 2**	**Animal 3**	**Animal 4**	**Animal 5**	**Animal 6**	**Total**	
0	8	11	4	15	14	6	58	
1	5	7	5	10	13	5	45	
2	4	7	8	2	8	6	35	
3	7	1	7	1	5	3	24	
4	13	1	3	2	1	6	26	
**B**	**Probability of Exhibiting Behavior**							
**State**	**Spec. Mon**.	**Spec. Rot**.	**Spec. Trans**.	**Gen. Mon**.	**Gen. Rot**.	**Gen. Trans**.	**Deimatic**	**Groom**
0	35% (24%)	20%(17%)	9%(11%)	24%(23%)	1%(4%)	4%(14%)	2%(7%)	3%(8%)
1	34%(26%)	15%(12%)	8%(12%)	33%(24%)	1%(5%)	1%(5%)	1%(4%)	7%(11%)
2	44%(22%)	9%(10%)	6%(11%)	34%(25%)	1%(1%)	0%(1%)	1%(4%)	6%(8%)
3	33%(22%)	9%(16%)	2%(4%)	43%(25%)	3%(5%)	2%(6%)	0%(1%)	6%(9%)
4	20%(23%)	4%(5%)	1%(2%)	42%(28%)	11%(15%)	9%(16%)	0%(2%)	7%(10%)
**C**	**Duration of Time Spent Exhibiting Behavior (seconds)**							
**State**	**Spec. Mon**.	**Spec. Rot**.	**Spec. Trans**.	**Gen. Mon**.	**Gen. Rot**.	**Gen. Trans**.	**Deimatic**	**Groom**
0	3.47(4.55)	1.21(1.28)	1.83(2.13)	4.99(7.08)	0.39(0.84)	0.72(2.48)	0.22(0.97)	1.79(5.35)
1	3.07(4.09)	0.84(1.15)	1.57(2.29)	6.07(6.33)	0.45(1.16)	0.11(0.54)	0.19(0.95)	3.25(4.20)
2	3.57(5.50)	0.46(0.41)	1.07(1.26)	8.53(9.60)	0.32(0.38)	0.03(0.18)	0.29(1.10)	3.27(4.22)
3	3.36(7.10)	0.38(0.36)	0.64(1.73)	9.91(10.79)	0.87(1.13)	0.69(1.14)	0.05(0.25)	5.59(6.12)
4	3.11(4.80)	0.41(0.54)	0.31(0.85)	11.49(17.26)	1.15(1.24)	1.39(1.61)	0.14(0.70)	7.93(11.88)
**D**	**Number of Transitions Into Behavior**							
**State**	**Spec. Mon**.	**Spec. Rot**.	**Spec. Trans**.	**Gen. Mon**.	**Gen. Rot**.	**Gen. Trans**.	**Deimatic**	**Groom**
0	245	246	85	85	61	58	58	60
1	233	255	66	87	53	45	45	58
2	313	310	46	98	45	35	35	48
3	201	229	26	130	59	28	24	37
4	187	195	29	120	75	30	26	32
**E**	**Misc. Statistics**							
**State**	**Avg Subseq Time (s)**	**Miss Strike (count)**	**Success Strike Rate**	**Prey Capture**	**Escape Attempts (count)**			
0	33.80(40.27)	33	73%	50/58; 86%	6			
1	39.84(39.64)	28	74%	43/45; 96%	1			
2	66.60(75.28)	25	66%	33/35; 94%	0			
3	98.03(129.53)	16	69%	20/24; 83%	1			
4	95.33(130.23)	9	37%	11/26; 42%	12			

### 2.3 Videographic data extraction: Frame-by-frame behavior definitions

Every video used in the data analyses was assessed by two independent experimenters for the frame-by-frame labeling of praying mantis behavior. Each video frame serves as a time step, *t*, which has an observed behavior exhibited by the praying mantis. The observed behavior at each time step is then assigned to one of the twelve pre-defined behavior bins delineated in the bin descriptions below. The results section of this paper also makes reference to a hunting regime versus a nonhunting regime. In this paper, the mantis behaviors that are directed *at* prey would be considered a *hunting-specific behavior* as it furthers the goal of prey capture. Behaviors that did *not* appear to further the effort of prey capture, would be considered a part of the *nonhunting*, general behavior regime. In the behavior bin explanations that follow, each behavior is noted to belong to either the hunting or nonhunting regime.

**Fig 1:**
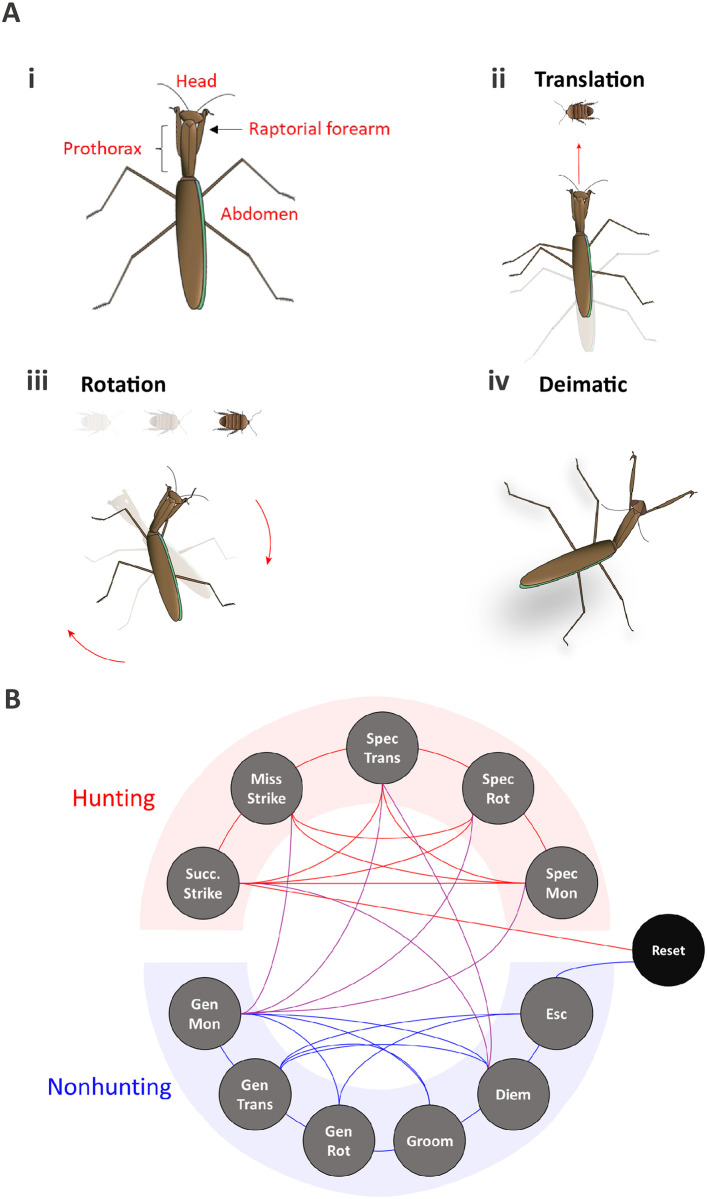
Examples of exhibited behaviors and behavior transitions. (Ai) Anatomical designations that may be references in the text with examples of the behavioral actions of translation (Aii), rotation (Aiii), and deimatic fear response (Aiv). (B) The freely behaving praying mantis can transition between the bins with few experimenter-defined constraints as outlined in the text.

1. **Successful strike**: Ballistic extension of the raptorial forearms (Fig 1Ai shows schematic of anatomy) in the attempted capture of a cockroach nymph. In our analysis, the strike is treated as an instantaneous behavior, meaning the number of frames to perform the strike was not taken into consideration. This was done for two reasons: animals in this experiment did not transition behaviors mid-strike, so the whole strike sequence was treated as a single, instant event. Secondly, exploring strike kinematics was not an objective of this work (see [51] for better overview of strike dynamics). The analysis of this paper is concerned with state dependent transitions so having striking behavior be instantaneous was sufficient. Of further note, the kinematics of striking behavior can be complex [19, 51]; sometimes the praying mantis is noted to lean into prey, lunge towards prey, or peer prior to the ballistic strike. These pre-strike behaviors were not counted as part of the strike itself, but rather were categorized into an appropriate action bin separate from the strike, such as specific monitoring (described below). This behavior is considered to be a hunting behavior.
2. **Missed strike**: Attempted capture of a cockroach but was unsuccessful. Like the successful strike, this was treated as an instantaneous behavior. This behavior is considered to be a hunting behavior.
3. **Specific translation**: Translation towards a specific nymph (Fig 1Aii). The praying mantis uses “specific translation” to close the distance to the prey, attempting to place herself within striking distance [52]. This behavior is considered to be a hunting behavior.
4. **Specific rotation**: Rotation of the head, prothorax, total body, or combination thereof towards a specific nymph (Fig 1Aiii). This behavior stems from the praying mantis’s tendency to centralize prey in the center of the visual field by updating her heading to maintain prey positioning [19, 49, 53–55]. This behavior is considered to be a hunting behavior.
5. **Specific monitoring**: Continued visual monitoring of prey that follows indicators of attention towards said prey (e.g. head turns, translations, or antennal positioning). This bin is inclusive of slight movements such as leaning towards prey and peering [56]. This behavior is considered to be a hunting behavior.
6. **General monitoring**: Stationary assessment of the arena *without specific attention* paid towards specific prey. This behavior is similar to specific monitoring except it does *not* follow indicators of attention. This behavior is considered a neutral or ambiguous behavior since it is not clear if whole-arena monitoring better serves the ultimate goal of prey capture or is a state of low-energy when not in the hunting mode. General monitoring is plotted with nonhunting behaviors to keep it distinct from the more aggressive hunting actions stated above.
7. **General translation**: Translation *not* towards specific prey. This behavior is considered to be a nonhunting behavior.
8. **General rotation**: Rotation of head, prothorax, total body, or combination of the above *not* towards specific prey This behavior is considered to be a nonhunting behavior.
9. **Grooming**: Cleaning of the raptorial arms, head, antennae, or legs [19, 51]. This behavior is considered to be a nonhunting behavior.
10. **Deimatic display**: Stereotyped defensive display towards a predator or threatening object, with forearms, wings, and/or abdomen raised in threatening posture (Fig 1Aiv) [19, 55]. This behavior is considered to be a nonhunting behavior.
11. **Escape**: Attempting to climb the experimental arena walls. This behavior is considered to be a nonhunting behavior.
12. **Arena reset**: Although not a behavior exhibited by the animal, an arena reset is an absorbing bin that signifies the end of a behavior sequence. This was a direct result of a behavior that necessitates the experimenter to intervene. For most experimental runs, the arena reset was usually prompted by the mantis capturing a nymph. Less often, arena resets occurred due to animal attempts at escape, and in one instance, the experimenter adjusted the scene to move the cockroach nymphs.

The small time step of video capture allows even subtle behavior transitions to be included in the analysis of freely behaving hunting mantises (Fig 1B). A single video, however, was not analyzed as a single continuous string of behaviors. As discussed in the “arena reset” bin description, certain behaviors would signal the end of a sequence. The most obvious example of an arena reset is a successful capture of a nymph within the 7 minute run. At this point, the experimenter removes the nymph from the grasp of the praying mantis and places both animals back into the arena. The placement of the mantis back into the arena signifies the beginning of a new sequence of behavior. These behavior sequences, separated by arena resets, are referred to as subsequences and a single video (i.e. experiment in a feeding state) can have multiple subsequences.

**Fig 2:**
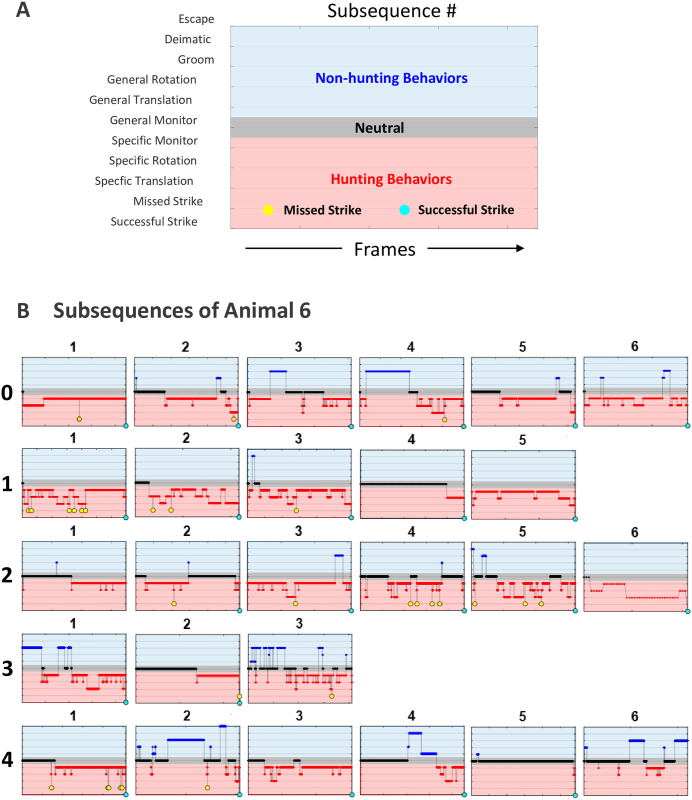
Example raw behavioral data across all feeding states (0-, 1-, 2-, 3- and 4-fed). (A) Color and symbol key for interpreting plots of part (B). The defined behaviors (y-axis) are plotted against frame number (x-axis). The colors red/black/blue are used to signify the different behavioral regimes. Red corresponds to the defined hunting behaviors: specific translation, specific rotation, and specific monitoring towards prey. Within the red hunting regime, strikes are denoted with circles; missed strikes are filled with yellow while successful strikes are cyan (which can only occur at the end of a subsequence as it will causes an arena reset). The grey region is the general monitoring where the praying mantis is motionless and not attentive to prey. Blue corresponds to the nonhunting behaviors: general translation, general rotation, escape attempts, grooming, and deimatic posturing. (B) Animal 6 exemplifies a time progression of behavior transitions across all feeding states. Here, the numbers to the left signify the feed states 0-, 1-, 2-, 3- and 4-fed. The behavior trace for each subsequence shows more hunting behaviors in the early feed states, 0-, 1-, 2-fed. Note that each subsequence ended in a successful strike save three. Subsequence 1 in the 2-fed state had an arena reset due to the experimenter readjusting the roach nymphs in the arena. Subsequence 3 in the 3-fed and subsequence 6 in the 4-fed state both end due to the end of the allotted 7 minutes. Each feeding state tended to have multiple subsequences of behavior.

Additional behaviors that resulted in resets include, escape attempts(the praying mantis attempting to climb the acrylic walls), and in one instance, specific monitoring due to the experimenter readjusting placement of the cockroach nymphs (Fig 2B 2-fed, subsequence 1). Lastly, the conclusion of the 7 minute trial concludes the current behavior subsequence, with the current behavior serving as the final behavior of the subsequence.

### 2.4 Analysis

Data matrices were organized using Excel (Microsoft, Seattle, WA) and plotted using Matlab (The Mathworks, Natick, MA). Statistical analysis of behavior time was done in JMP (JMP Pro 5.0.0 SAS Institute Inc., Cary, NC, 1989-2019.). Sequence analyses were done in Matlab with custom scripts.

## 3 Results

### 3.1 Behavior sequences

To provide a qualitative look at the raw behavior transitions, Fig 2B shows how one of the experimental mantises (animal 6) occupied behavior bins for each time step across all feeding states. As the animal becomes more sated, it has fewer subsequences dominated by red regime behaviors (hunting) and a higher proportion of both blue (nonhunting) and grey (general monitoring) behaviors. Additionally, the number of attempted captures (i.e. missed strikes) decreases as seen by fewer yellow circles appearing in the sated subsequences. With this qualitative assessment of behavior, it seems reasonable that perhaps the animal does transition from hunting-focused behavior when in starved conditions to a nonhunting regime as motivation changes.

### 3.2 Behavior time

The first metric considered is time and we explore this concept from two standpoints: first, does nutritional status correlate with the *probability* of exhibiting a behavior within a subsequence, and secondly, does satiety correlate with the *duration* of time in which an animal remains in a single behavior? These two questions are similar but allude to subtle differences that are discussed in the sections below.

#### 3.2.1 Behavior probability

**Fig 3:**
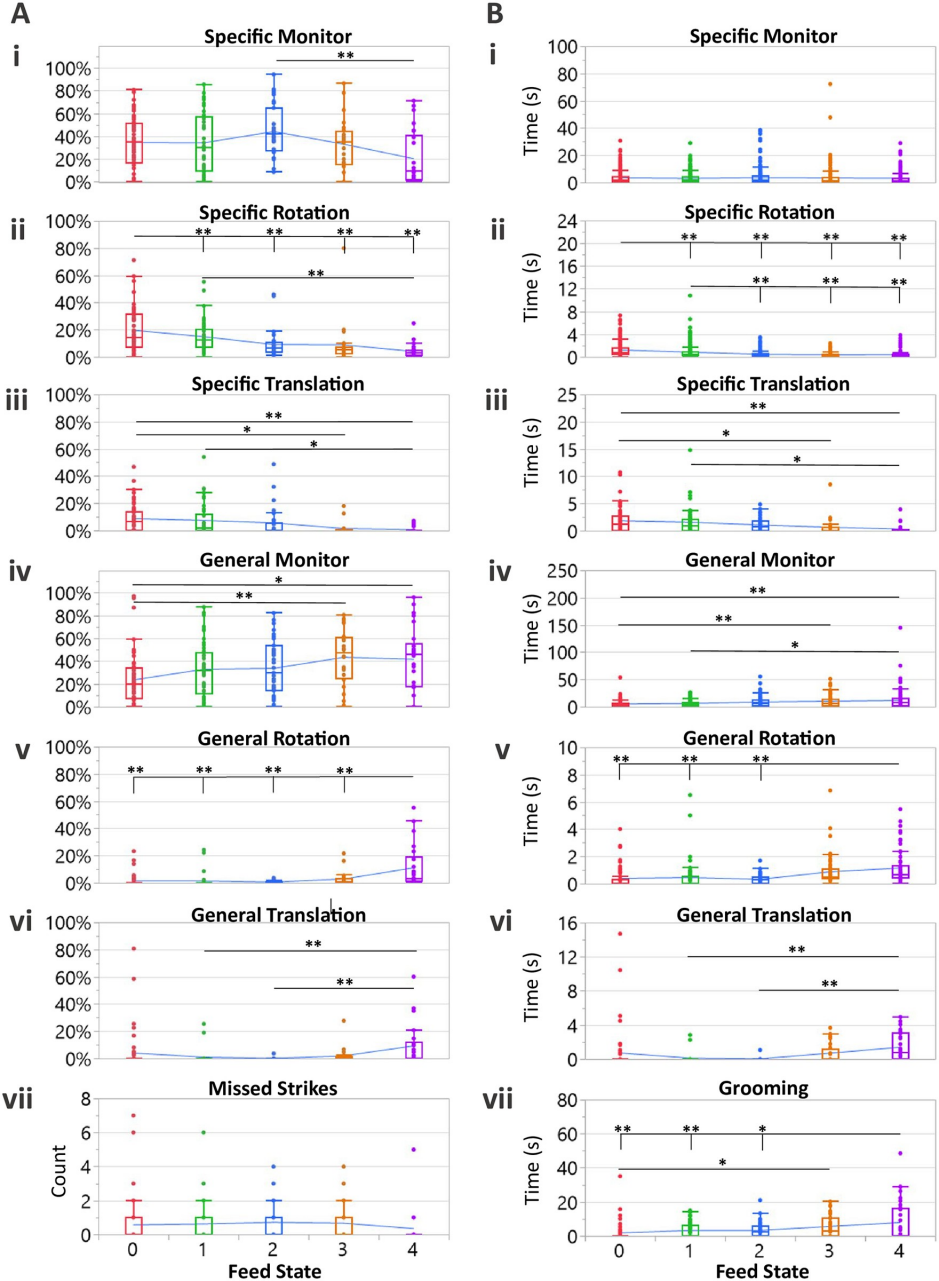
Behavioral probability and bout time analyses across all feeding states. (A) Probability plots for hunting behaviors exhibited by feed state. (Ai) Specific monitoring shows a slight increase in the 2-fed animals with a statistical difference relative to the 4-fed state (ANOVA: p = 0.0054). Decreasing probabilities of both specific rotation (Aii) and translation (Aiii) as satiety increases (ANOVA: p <0.0001 and p = 0.0018, respectively). (Aiv) Number of missed strikes per subsequence across feeding states. Missed strikes showed a decrease for the 4-fed state (non-statistical trend; ANOVA: p = 0.78). Trends show increased probability of both general monitoring (Av), general rotations (Avi), and general translation (Avii) as satiety increased (ANOVA: p = 0.0036, p <0.0001, and p = 0.0048). For (Ai)—(Avii), N = 58, 45, 35, 24, 26 which is equal to the number of subsequences exhibited in each feed state. (B) Bout time plots for hunting behaviors exhibited by feed state. (Bi) Specific monitoring bout times were consistent across feed states (ANOVA: p = 0.79). A downward trend was seen for both specific rotation (Bii) and specific translation (Biii) across the feed states (ANOVA: p<0.0001 and p = 0.0008, respectively). An upward trend in bout time was seen for general monitoring (Biv), general rotation (Bv), general translation (Bvi), and grooming (Bvii) as the feed state increased (ANOVA: p = 0.0002, p<0.0001, p = 0.0029, p = 0.0002, respectively). For part (B), N for each behavior is reported in Section D of Table 1. Tukey pairwise comparisons were performed when ANOVA analysis produced a pValue < 0.05; Tukey pValues: *p<0.05 and **p<0.01.

For each subsequence, the number of frames (i.e. time) spent in each behavior was divided by the total time length, thus producing a probability of observing each behavior. Specific monitoring probabilities were similar across all feeding states with the exception of the 2-fed state that showed a slight increase in probability of exhibiting this behavior (Fig 3Ai; Section B in Table 1; ANOVA: p = 0.0054). Specific rotation and translation both show a downward trend in these aggressive behaviors as a function of increasing satiety (Fig 3Aii and 3Aiii, respectively; Section B in Table 1; ANOVA: p < 0.001 and p = 0.0018, respectively; Tukey pairwise comparisons are noted in the caption).

Conversely, nonhunting behavior probabilities were a more dominant feature in sated states, with all nonhunting behaviors showing a statistical increase as satiety increased (Fig 3v–3vii; Section B in Table 1; all ANOVA analyses: p < 0.05; Tukey pairwise comparisons are noted in the caption). Note that escape attempts are not reported as probabilities, but rather the number of attempts. This was necessary due to that fact that the time spent in the escape behavior can vary depending on how quickly the experimenter stops the escape attempt to reset the arena. The data shows a sharp increase in the 4-fed state, however, almost all escapes can be attributed to a single mantis, animal 1. This animal made 6 escape attempts while in the 0-fed state, 1 escape attempt in both the 1- and 3-fed states, and a total of 8 attempts in the 4-fed state. Outside of animal 1, escape behavior was very rare (Section E in Table 1).

Like many of the behaviors, both successful and missed strikes showed trends with hunger level as well. The percentage of subsequences that were concluded with successful strikes and thus prey capture was rather similar across 0-, 1-, 2-, and 3-fed animals, with a dramatic drop off in prey capture seen in the 4-fed state (Section E in Table 1; *χ*^2^(4, *N* = 188), p < 0.001). This was in part due to fewer *attempts* at prey capture in the more sated animals. The number of missed strikes exhibited in each subsequence across feed states was not significantly different (Fig 3Aiv; Section E in Table 1; p = 0.78). With fewer attempts and fewer successes, unsurprisingly, the success rate showed a sharp decrease in the 4-fed state (Section E in Table 1).

The data in this section shows that the low nutritional states (0- and 1-fed) exhibited a hunting-specific predominance with 64% and 57% probability of being in hunting behaviors, respectively. Counter to this, 3- and 4-fed animals had a probability of 44% and 25% of being in a hunting behavior. The 2-fed state shows a shift, where 2-fed animals spent an overall 59% of behavioral time in the hunting regime but using less time in actionable behaviors like head/body turns (9%) or closing the distance to prey with translation (6%). Rather, animals in this feeding state had increased vigilance towards prey items (44%), while not investing energy into pursuit.

#### 3.2.2 Behavior duration

The analysis here is similar to that presented in section 3.2.1, but in this section, the question being asked is how *long* does the animal remain in a behavior once that behavior is initiated? Fig 3B summarizes these results and highlights the differences across feeding states in how behaviors are sustained once initiated.

Animals in the lower satiety states tended to remain in specific rotation and translation longer than sated animals, which is reflective of the fact that starved animals tracked and pursued prey even when large angles or distances separated predator and prey (Fig 3Bii and 3Biii, respectively; Section C in Table 1; ANOVA analyses: p < 0.05 for both plots). This result is concordant with the work done by Bertsch et al. who mapped a relatively larger area of attention to starved animals and found them to be more predatory to more distant prey. Inversely, time spent in the nonhunting behaviors of general rotation and translation increases with an increase in satiety, denoting the willingness of sated animals to utilize nonhunting behavior for longer bouts of time while in the presence of food items (Fig 3Bv–3Bvi; ANOVA analyses: p < 0.05 for all plots) [20].

Both general and specific monitoring entail this seemingly “passive” assessment of the environment, however, specific monitoring appears to be implemented with consistent bout time when the animal is actively in a state of attention—regardless of feed state. Fig 3Bi shows that specific monitoring time *duration* did not strongly trend with the nutritional status, suggesting a threshold of allowable idle time (Section C in Table 1; ANOVA analyses: p > 0.05). This is in contrast to general monitoring in which bout time was positively correlated with feeding states (Fig 3Biv; Section C in Table 1; ANOVA analyses: p < 0.05).

### 3.3 Sequence analysis

Sequential data is a class of data in which the order of the outputs becomes a very relevant aspect in modeling the given system [57–59]. For example, language relies on the ordering of symbols in specific sequence structures such that embedded meaning can be conveyed [60]. When comparing entire sequences for similarity, specialized approaches are needed such that the sequential ordering of data is not obscured. This section of the analysis approaches the behavior sequences of the praying mantis from the assumption that patterns may in fact exist, not only in the time spent in each behavior as discussed above, but also in the *order* in which these behaviors present. The following subsections explore different aspects of sequential behavior analysis to draw insight into whether exhibited behavior of the praying mantis takes on different characteristics as the feeding state changes.

#### 3.3.1 Transition probabilities

**Fig 4:**
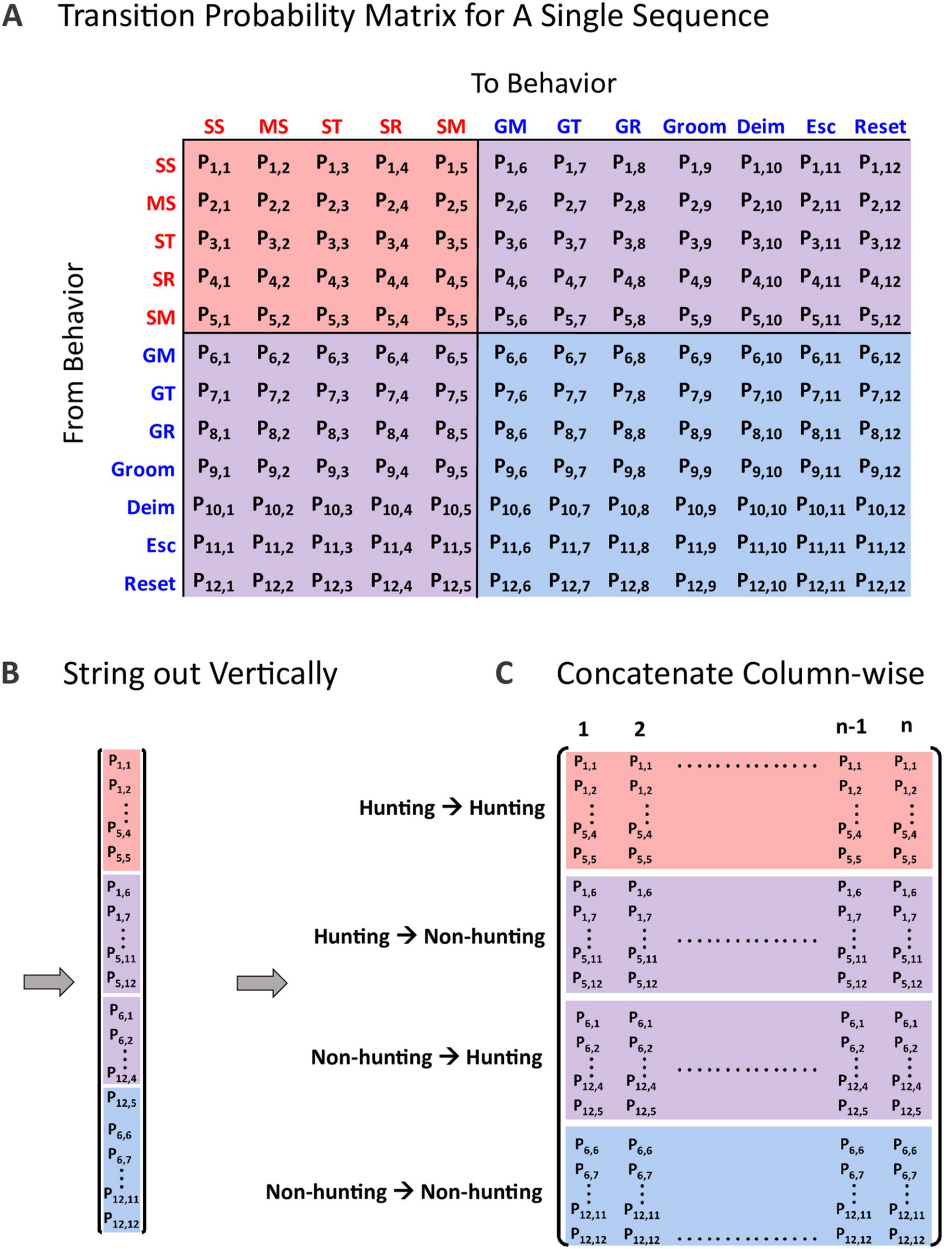
Transition Probability Matrix. (A) Example organization of the square transition probability matrix. The probability, *P*, of transitioning out of behavior *i* to behavior *j* is held in the matrix element, *i*,*j*. For example, *P_3,1_* represents the observed probability of the animal transitioning from behavior 3 (specific translation, ST) to behavior 1 (successful strike, SS). The color coding seen in (A) denotes the regime in which each behavior belongs (red = hunting regime behaviors; blue = nonhunting behaviors). The red shaded region of the square matrix corresponds to the probabilities of hunting behaviors further transitioning into hunting behavior. The purple regions show the transition probabilities corresponding to a regime change; this either occurs when there is a transition from a hunting behavior to a nonhunting behavior, or conversely, when transitioning from a nonhunting behavior to a hunting behavior. The blue shaded region signifies transitions that remain in the nonhunting regime. To best show many transition matrices across all feed states in a single figure, the 12 x 12 matrix for each subsequence was reorganized as a column vector. (B) The column vector holds all the probabilities from the square matrix, and is structured according the color coding seen in the figure. (C) After each transition matrix corresponding to each subsequence is converted to a column vector, all the column vectors can be concatenated into a single, 2D matrix, with the maintained shaded regions. SS = successful strike; MS = missed strike; ST = specific transition; SR = specific rotation; SM = specific monitoring; GM = general monitoring; GR = general rotation; GT = general translation; Groom = grooming; Deim = deimatic; Esc = escape.

By assembling the behavior in each time step, a transition probability matrix can be assembled for each subsequence (i.e. sequence A will produce a 12 x 12 transition matrix, *T*_*A*_ while sequence B will produce its own corresponding 12 x 12 transition matrix, *T*_*B*_). This is a square matrix that holds the observed probabilities of each behavior transitioning into every other behavior during a subsequence (Fig 4A). To provide a qualitative assessment of all the transition matrices as a correlate of satiety, the square matrix shown in Fig 4A is strung out vertically into a “feature vector,” where each row is reorganized and stacked vertically into a single column vector in accordance to Fig 4B). With the probability matrix for each subsequence reconfigured to a column vector, the vectors were grouped in order of increasing satiety (e.g. all 0-fed column vectors grouped side-by-side; Fig 4C).

**Fig 5:**
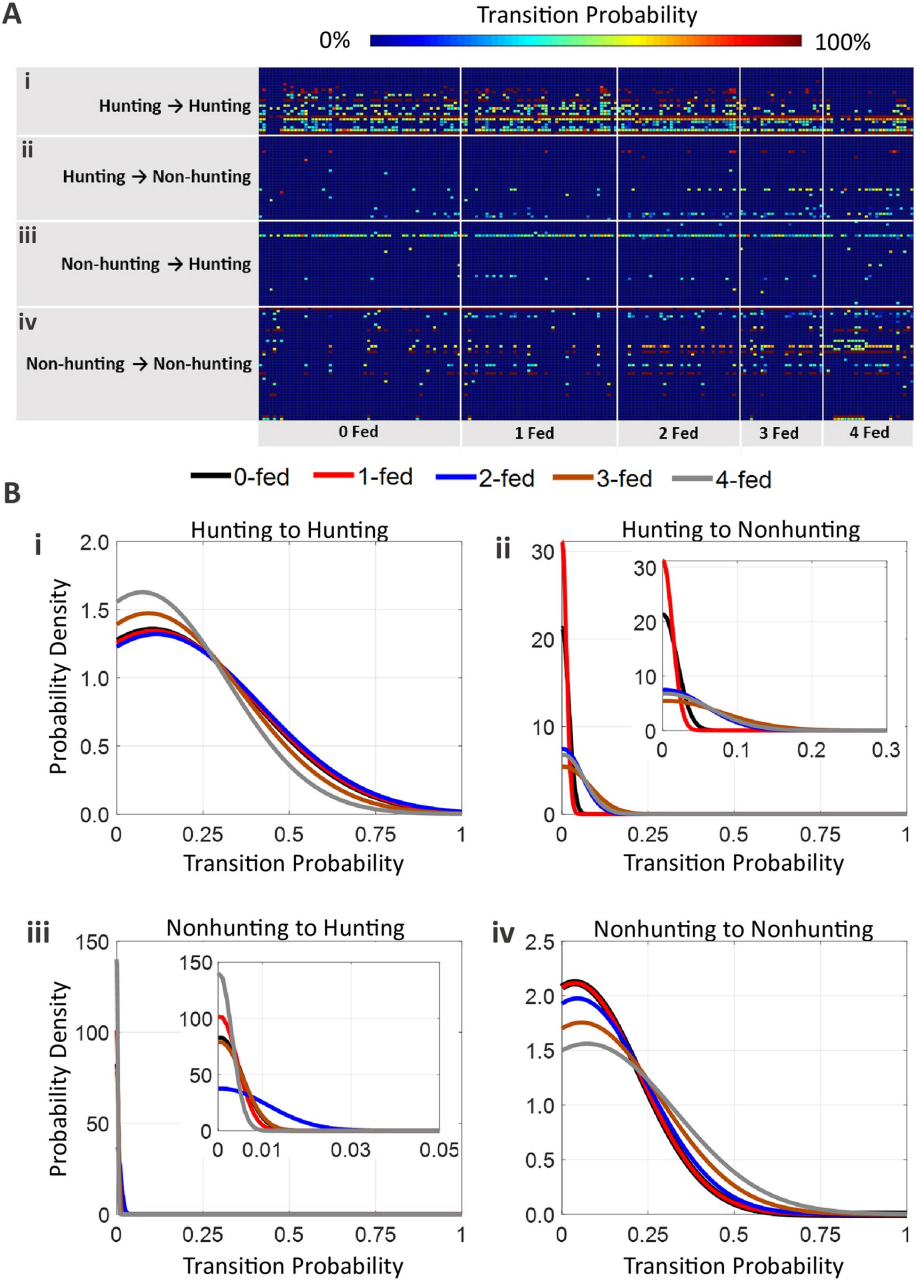
Transition probability trends across feed states. (A) Probability heat map of transition probabilities across all feed states and organized to show trends among the different transition types: Hunting→ Hunting (i), Hunting→ Nonhunting (ii), Nonhunting→ Hunting (iii), and Nonhunting→ Nonhunting (iv). Dark blue areas correspond to very low probabilities of seeing the transition, while non-blue colors correspond to higher probabilities of state transitions occurring. (B) Quantifies the trends seen in the heat map of part (A). The probability density plots show distribution of transition probabilities that were exhibited across all feed states for each transition type: Hunting→ Hunting transition (i), Hunting→ Nonhunting transition (ii), Nonhunting→ Hunting transition (iii), and Nonhunting→ Nonhunting transition (iv). The area under each curve in the density plots is equal to one, however, how the area is distributed across the probabilities signifies the density. Probability values with higher density means higher frequency of those values in the transition probability matrices.

The processing steps of Fig 4 allow the transition matrices to be quickly compared across all feeding states. Applying color-coding to the values found in the probability vertical matrices from Fig 4C generates the heat map in Fig 5A. Looking at the hunting → hunting region (Fig 5Ai), 0-fed and 1-fed states show a dense scattering of colored cells of non-zero transition probabilities, indicating that for low feeding states, hunting behavior tended to lead into further hunting behaviors and that starved animals will predominately remain in the hunting regime. Conversely, as satiety increases across Fig 5Ai, 2-, 3-, and 4-fed animals show progressively sparser scatterings of colored cells, meaning these feeding states did not promote consecutive hunting behaviors as often. This is further supported by the density plot of hunting → hunting transition probabilities in Fig 5Bi. In this density plot, the lower feed states show a low density of smaller probabilities and a higher density of large probabilities, meaning that starved mantises are more likely repeatedly transition within the hunting regime, and by extension, not exit hunting behavior. Fig 5Bi also shows the reverse is true for sated states, where the larger densities of small probabilities means the sated animal is less likely to remain in hunting.

Similar correlations to feed state appear across both hunting → nonhunting (Fig 5Aii) and nonhunting → nonhunting transitions (Fig 5Aiv), where both show increasing colored cells as satiety increases. Progressively wider density distribution are seen on the density plot for hunting → nonhunting transitions (Fig 5Bii), suggesting an increasing tendency of sated animals to transition *out* of hunting and into nonhunting behaviors. Fig 5Biv shows the same trend in density distribution, indicating that sated animals currently in nonhunting behavior remained in nonhunting behavior more often.

Fig 5Aiii and 5Biii show the transition probabilities for nonhunting → hunting, which do not show a clear trend. The density plot of Fig 5Biii shows an overwhelming density concentration around zero for all feed states; zooming in on this region, all feed states had below a 1% chance of exhibiting nonhunting → hunting transitions, except the 2-fed state for which this transition type slightly more likely.

**Fig 6:**
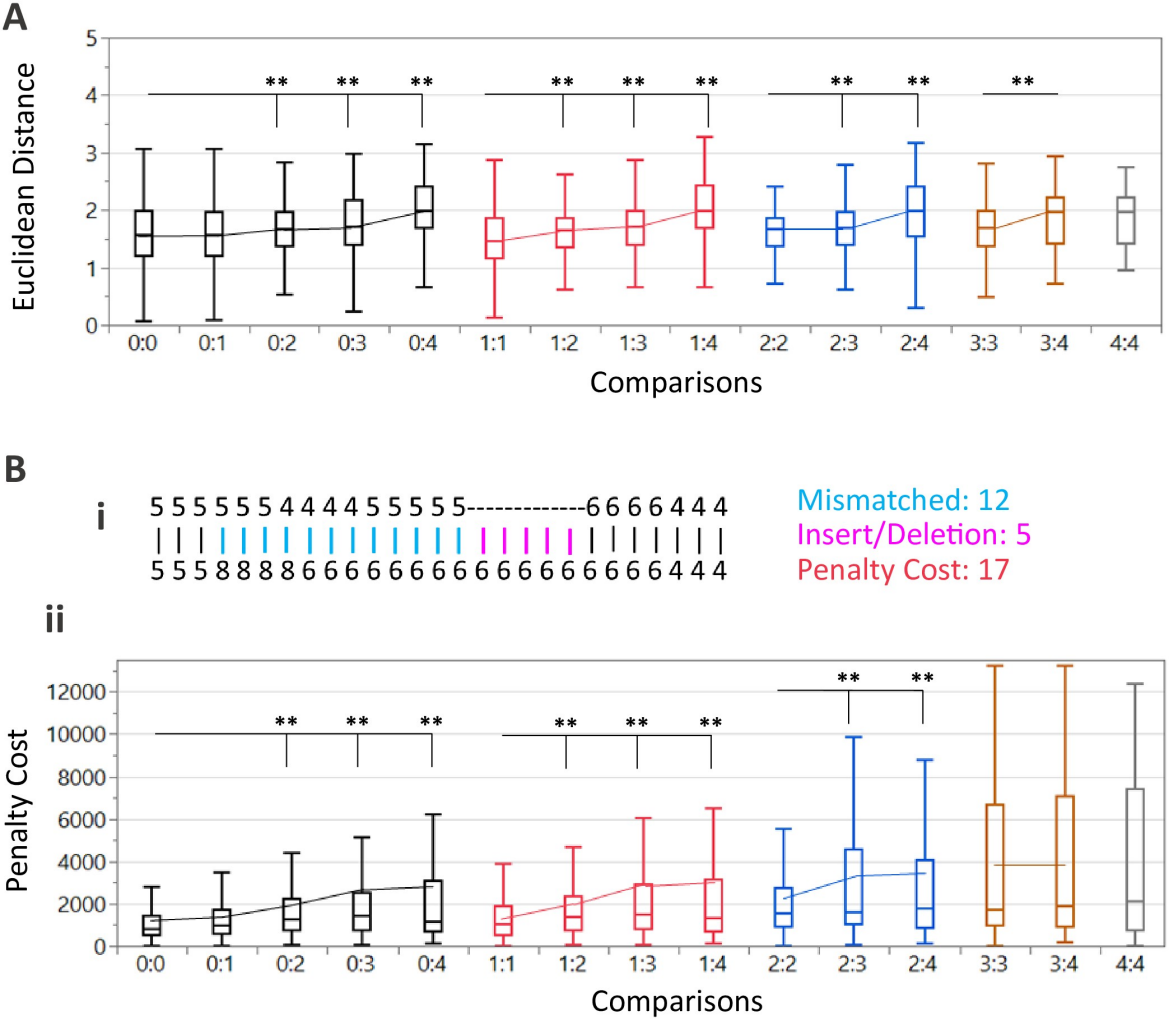
(A) Euclidean distances between transition probability matrices for each feed state. Box plot colors (black, red, blue, orange, grey) are used to help focus trends in specific comparisons groupings, while the line is connecting each box plot *mean*. For example, the black boxes are showing that each feed state exhibits an increasing Euclidean distance away from 0-fed, indicating a greater difference in transition probability profiles. The upward trend seen for the black grouping means that as satiety increases, behavior transitions progressively increased in dissimilarity when compared to the 0-fed state. All the colored groupings show similar trends, with ANOVA analysis showing a pValue < 0.05 for all groups. Tukey pairwise comparisons are shown: *p<0.05 and **p<0.01. (B) Penalty cost as a metric of sequence structure similarity. (Bi) Demonstration of PC calculation showing the cost incurred to edit a mismatch and insert/deletion. (Bii) Penalty cost from each feed state was compared to all other feed states. As was the case with (B), the connecting line in (Bii) is connecting each box plot *mean*. Black, red, and blue groupings show upward trends in the PC, indicating that sequence structure progressively changes as satiety increases (ANOVA analysis showing a pValue < 0.05 for these groups). Tukey pairwise comparisons are shown: *p<0.05 and **p<0.01. No statistical difference was seen in structure when comparing the 0- to 1-fed sequences, nor when comparing the 3- to 4-fed sequences.

To further quantify these apparent differences in transition probability matrices, the Euclidean distance [61] was calculated between each probability matrix from every feed state and the resulting distance distributions plotted in Fig 6A. With this approach, the probability vectors occupy a data space, where the vectors belonging to a single feeding state would constitute a cluster of high dimensional “points.” The distances measured within this cluster would represent the spread of these points belonging to the particular feeding state. When the vectors from remaining feeding states are projected into the same data space, distance distributions can also be generated between projections and give a *relative* metric of probability matrix similarity—with greater similarity indicated by smaller distances. Comparing across all feeding states, the distance *means* between transition probability vectors consistently show statically significant increases (i.e. progressively greater dissimilarity) as satiety increases.

When making comparisons across feed states in Fig 6A, colored box plot groups were used to help focus key trends. For the black box grouping, the 0-fed state serves as a reference to be compared to all other feed states. The x-axis shows what feed states are being compared, with the notation 0:0 meaning the distance was calculated between all 0-fed transition probability vectors to every other 0-fed vector. Likewise, 0:4 corresponds to the distances calculated between all 0-fed vectors and 4-fed vectors, and so on. The line connecting the black boxes goes through each box mean, and exhibits an upward trend. This positive slope indicates that higher feed states produce probability vectors that are are increasingly different from the 0-fed transition probability vectors. For the two low feed state comparisons (0:0 and 0:1), however, transition profiles did not statistically differ (Tukey pairwise mean comparison: p > 0.05). In other words, the *intra*state distance distribution (mean and standard deviation) seen when 0-fed vectors are compared to all other 0-fed vectors (0:0) was comparable to the distance distribution calculated between 0-fed and 1-fed vectors (0:1). This indicates that both of these nutritional states produced similar behavioral transitions, while increasing satiety (2-, 3-, and 4-fed) correlates with progressively differing transition probability profiles from the food deprived states (0- and 1-fed).

The subsequent color groupings continue this trend, with the red grouping showing higher feed states against 1-fed as the reference, the blue grouping against 2-fed as the reference, and the orange grouping with 3-fed as a reference. These three groupings, red, blue, and orange all show increasingly different transition profiles of higher feed states when compared to the group reference.

#### 3.3.2 Editing penalty cost

The editing penalty cost (PC) (also called the Levenshtein distance or the editing distance) is a metric that has been used across a number of disciplines such as proteomics [62], genetics [63], and vocalization sequence analysis [64] and aims to quantify similarities in sequence structure. This approach does a pairwise comparison between two subsequences, *A* and *B*, and calculates the total number of edits needed to make the two sequences match [58, 61, 65, 66]. This distance metric is in fact the “cost” incurred to do such editing operations as single symbol edits (at loci of mismatches) and single symbol deletions/insertions at loci where the longer sequence does not have a corresponding overlap from the shorter sequence [67].

Fig 6Bi shows an example schematic outlining this process; when two sequences *A* and *B* are compared, the two sequences are initiated with the maximum match overlap possible—even if the shorter sequence is segmented to achieve this maximum overlap. Looking at the example, mismatches are loci of symbol overlap that do not match (shown in blue of Fig 6Bi). If the top sequence were to have twelve of its symbols (‘555444455555’) replaced with ‘888866666666’ (or vice verse), the two sequences would be better paired at the cost of twelve edits. In the case where a shorter sequence is segmented to maximize matching, gaps will overlap with the symbols of the partnering sequence (shown in pink of Fig 6Bi). In these gaps, the insertion of five symbols, ‘66666’, would also improve the pairing of the sequences at a cost of 5 edits. Such edits are done down the length of the two sequences until they are perfectly matched. The penalty cost incurred to achieve a perfect matching totals to 17 in this example.

Like in the data presentation used in section 3.3.1, color groupings are used to help focus comparisons made in Fig 6Bii. The black box grouping shows the penalty cost between each vector from all feed states and the 0-fed state reference. The connecting line between the boxes passes through the distribution mean of each pairing; the upward trend of this line signifies that increasing feed states produces progressively different behavior sequences from that of the lower feed states (0- and 1-fed). Like the results seen in the transition probability vectors distances, 0-fed and 1-fed produce similarly structured behavior sequences (Tukey pairwise mean comparison: p > 0.05). The subsequent color groupings, red and blue, both show this upward trend, further supporting that higher feed states exhibited different ordering of behavior than food deprived animals.

While the *inter*-feed state comparisons discussed above showed behavior structure differences between the various satiety states, the *intra*-feed state comparison shows how well behavioral sequence structure is preserved when the feed state is held constant (i.e. 0:0, 1:1, 2:2, 3:3, 4:4). The PC distributions for the *intra*-feed state comparisons 0:0, 1:1, and 2:2 are quite small and not variable. This indicates that within these lower feed states, behavior structure remains preserved throughout an experimental condition. This observation deviates for the higher feeding states where both 3:3, and 4:4 similarly have a large spread in PC values—indicating these feeding states present with more flexibility in sequence structure.

## 4 Discussion

The results of this paper further supports the work by Bertsch *et al*. that nutritional state greatly influence praying mantis predatory tactics [20]. Approaching hunting behavior as a process that can transition between discrete states allows the use of Markov chain analysis to be quite insightful. Transition probability matrices generated for each subsequence of behavior showed starved animals had a greater propensity to transition between hunting specific behaviors and did not often deviate from their hunting mode. This is in comparison to the transition profiles exhibited by sated animals, where transitions *out* of hunting were more common.

By utilizing sequential analyses, we showed that starved animals exhibited stereotyped sequences that adhered strongly to the goal-driven objective of prey capture. This conclusion is supported by the penalty cost results (section 3.3.2) that showed conserved behavior patterns across the two lowest feeding states (0- and 1-fed). These conserved behavior sequences of the 0- and 1-fed animals exhibited both a higher frequency of hunting→hunting transitions and longer duration of hunting-specific behaviors once they were initiated (sections 3.2.1–3.3.1). Specifically, this regime observed in the starved conditions was dominated by attending to prey and locomotion. Unexpectedly, grooming was observed as a behavior in this hunting regime, albeit with shorter epoch lengths. This suggests that quick cleaning of the raptorial forearms and antennae between hunting bouts likely provide a benefit to the praying mantis.

At the intermediate 2-fed state, there is deviation from the trends seen in 0- and 1-fed states. Here, the animal has a reduced utilization of pursuit and rotational tracking but maintains, however, a high level of specific monitoring. This suggests that there is a threshold of satiety where attention is still directed at prey but motivational shifts are beginning to digress from prey capture. This thresholding is also reflected in the penalty cost, where the structure of 2-fed sequences are distinct from that of both the starved states (0- and 1-fed) and sated states (3- and 4-fed).

As satiety increased, a greater variability of sequence structure emerged, suggesting that behavior becomes more fluid as the animal approaches nutritional competency. This is further supported by the transition analysis that shows stated animals to have a greater variety of transitions both into and out of hunting. These results allude to shifts in the hierarchy of behavioral expression. In desperate times when the survival of the animal is at stake (e.g. starvation), a robust suite of behaviors are expressed to stabilize the state of the animal. As the necessity subsides, the animal enters into a more plastic regime that permits a greater expression of variable behavior.

### 4.1 Unexpected use of behaviors

Grooming was originally hypothesized by the authors to be a behavior that would more commonly be seen in the sated state which is only partly true. Although the probability of observing grooming did not show a statistical trend across feeding states (ANOVA: p = 0.21), the *duration* of these grooming bouts did (ANOVA: p < 0.001). This implicates grooming as a generally important role regardless of behavior regime, although shorter bouts of grooming were seen in starved animals. This corroborates findings by Prete et al., where hungry animals were more attentive to eye debris with faster response times and fewer grooming cycles—constituting a shorter durations in grooming [19].

The use of deimatic responses, although not statistically significant, did appear more often in starved animals. Qualitatively, this is potentially due to the more aggressive nature by which starved animals navigated the arena—being more responsive to both prey and experimenter. Some of the deimatic displays were in response to the spatula used to nudge static nymphs from the corners of the arena and prompt them to scurry. An important point is that starved experiments were always conducted first and the increased use of the startle response could be attributed to sensitisation of the praying mantis to a perceived threatening stimulus. Although possible, we think the latter is less likely since the praying mantises were regularly handled by experimenters in the rearing phase prior to the start of experiments. In either case, deimatic displays were rarely deployed and constituted a very small portion of behavior, regardless of nutritional state.

### 4.2 Future work

Future work will explore the Markovian nature of the mantis behavior to quantify time dependencies of behavior repertoires. Markov modeling is a useful approach to discerning order-dependent trends within a data sequence. Built on the principles of dynamic Bayesian networks, Hidden Markov models (HMM) are a slight variation to the generic Markov modeling approach and aim to deduce the latent variables in the system [57, 58]. The latent variables are hidden states of the animal that are not directly evident (e.g. feed state) and tend to represent internal drivers of output behavior. Each hidden state has associated probabilities of producing observable behaviors (i.e. emission probabilities) and if given a sequence of behavior, dynamical programming can be used to recursively infer the most probable hidden state responsible for producing such an output [57–59]

Specific to this work, HMMs could be used to help quantify differences in output behavior sequences that belong to each feeding state. In this paper, changes in behavior sequence *structure* was shown to be correlated to feed states of the animal (sections 3.3.1 and 3.3.2). With an HMM approach, discerned patterns within the behavior sequences (i.e. features) are used as predictors of the most probable underlying feed state (i.e. class), allowing for finer resolution analysis of sequence *composition* changes that track with satiety. Through this type of analysis, it is our hope to help elucidate the role of internal states in the generation of complex, time sequenced behavior.
